# One Health, One Planet: rethinking public health in the era of pesticides, fertilizers, and antibiotics

**DOI:** 10.3389/fpubh.2026.1806971

**Published:** 2026-04-01

**Authors:** Azza Siddig Hussien Abbo, Marmar A. El Siddig

**Affiliations:** 1 Ministry of Municipality, Doha, Qatar; 2 Department of Crop Protection, Faculty of Agriculture, University of Khartoum, Khartoum North, Sudan; 3 Department of Botany, Faculty of Science, University of Khartoum, Khartoum, Sudan; 4 Faculty of Medical Sciences, Biosciences Institute, Newcastle University (NUBI), Newcastle upon Tyne, United Kingdom

**Keywords:** AMR, food contamination, modern agriculture, One Health, policy implications

## 1 Introduction

Through scientific and technological innovation, modern agriculture has developed into a key factor in determining public health, going far beyond its traditional role of guaranteeing caloric sufficiency to include food quality, nutritional security, disease prevention, and environmental sustainability (1). By affecting dietary composition, exposure to chemical hazards, and the ecological conditions that control disease emergence and transmission, agricultural systems have an impact on human health outcomes (2). Intensified production has significantly increased the amount of food available worldwide, but it has also resulted in environmental externalities that directly and indirectly threaten human wellbeing, such as soil degradation, water contamination, biodiversity loss, and ecosystem disruption (3).

This dual function draws attention to a basic paradox: agriculture both supports and hinders public health. A shift toward sustainable food systems that can strike a balance between ecological integrity and productivity is necessary to resolve this contradiction. In addition to promoting safety, nourishing food and lowering health risks related to chemical inputs, zoonotic diseases, and antimicrobial resistance (AMR), such systems must protect environmental resources (3, 4, 45).

## 2 Drivers of change in modern agricultural systems

### 2.1 From yield maximization to health-oriented food production

In the past, agricultural success was determined by maximizing yield, especially during the Green Revolution in the middle of the 20th century. Between the 1960s and 1980s, global food production doubled due to more productive crops, synthetic fertilizers, pesticides, and mechanization (1, 5, 6). Although these improvements decreased hunger in many areas, they frequently came at significant expense to the environment and public health. Pesticide residues, nutrient-poor diets, a decline in biodiversity, and long-term health risks such as cancer and endocrine disruption were all consequences of excessive chemical inputs (1).

As a result, the production of wholesome, safe, and health-promoting food has gradually taken precedence in agriculture. Modern methods, such as integrated pest management, organic farming, agroecology, and precision agriculture, seek to minimize contamination, increase nutrient density, and lessen reliance on chemicals without sacrificing productivity. The Sustainable Development Goals of the United Nations, especially those related to food security, health, clean water, and environmental protection, are closely aligned with this shift (7).

### 2.2 Socio-environmental and policy drivers

This paradigm shift has been accelerated by several of factors, such as population growth, fast urbanization, climate change, and growing consumer awareness of the health risks associated with food. These pressures have highlighted the vulnerability of food systems to disease outbreaks and environmental shocks, as well as the shortcomings of yield-centric agricultural models. However, the shift to sustainable agriculture is still uneven, hampered by fragmented regulatory frameworks, technological disparities, and financial obstacles for smallholder farmers (8, 9). Therefore, maintaining this transition requires effective policy integration, investment in innovation, and cross-sectoral cooperation.

## 3 Impacts of modern agricultural practices on health and the environment

The human species faces one of its most daunting challenges: how to produce enough food for the expected two billion additional people who will join the earth by 2050, and how to do this as climate change multiplies both biotic and abiotic threats to our food crops (10). Ancient farmers used to adopt natural selection methods to obtain seeds and fruits with specific characteristics for cultivation in the following seasons. Despite this, plant breeders are in an urgent and permanent need to improve the productivity of plants, improve the quality of their products, and give them protection qualities against biotic stress such as diseases and abiotic stress such as drought (11). Nature has demonstrated its remarkable efficiency in developing destructive dangers that target our food production. This is evident from the occurrence of more than twelve widespread outbreaks of plant diseases in the last century, affecting crucial food crops such as wheat, corn, rice, and potatoes, as well as other essential staples, fruits, and vegetables (12). The resulting attacks and the subsequent loss of food put the wellbeing and nourishment of millions of people at risk, while also causing significant financial burdens for farmers and consumers, amounting to billions of dollars annually (12, 13).

### 3.1 Pesticides and human health risks

**Figure 1:**
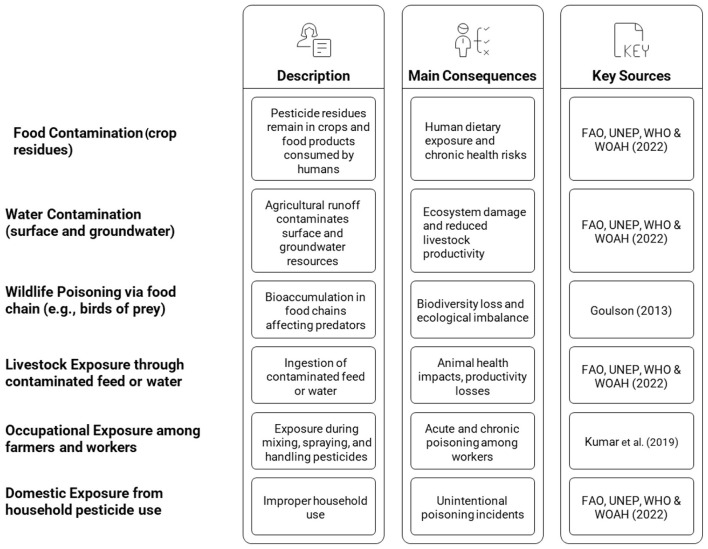
Major pathways of pesticide exposure affecting the One Health system.

Pesticides are still necessary for the protection of crops; however, their uncontrolled and abusive use has been fraught with risks which can endanger ecosystems and human health. Food and water contamination with pesticides residues is an important public health issues, as it affects animals too. For example, the poisoning of birds of prey can occur through pesticides used in food systems. Livestock can be exposed to hazards in feed and water. The contamination of water, for instance, may cause productivity losses. Unintentional poisonings, mainly arising from excessive exposure to and inappropriate use of toxic chemicals. including pesticides present in occupational and/or domestic environments, are heavily affecting human health, particularly in low-income countries. Exposure to mycotoxins, aflatoxins, biotoxins and waterborne pathogens is another problem of concern affecting the health of humans, animals and plants (Figure 1).

**Figure 2:**
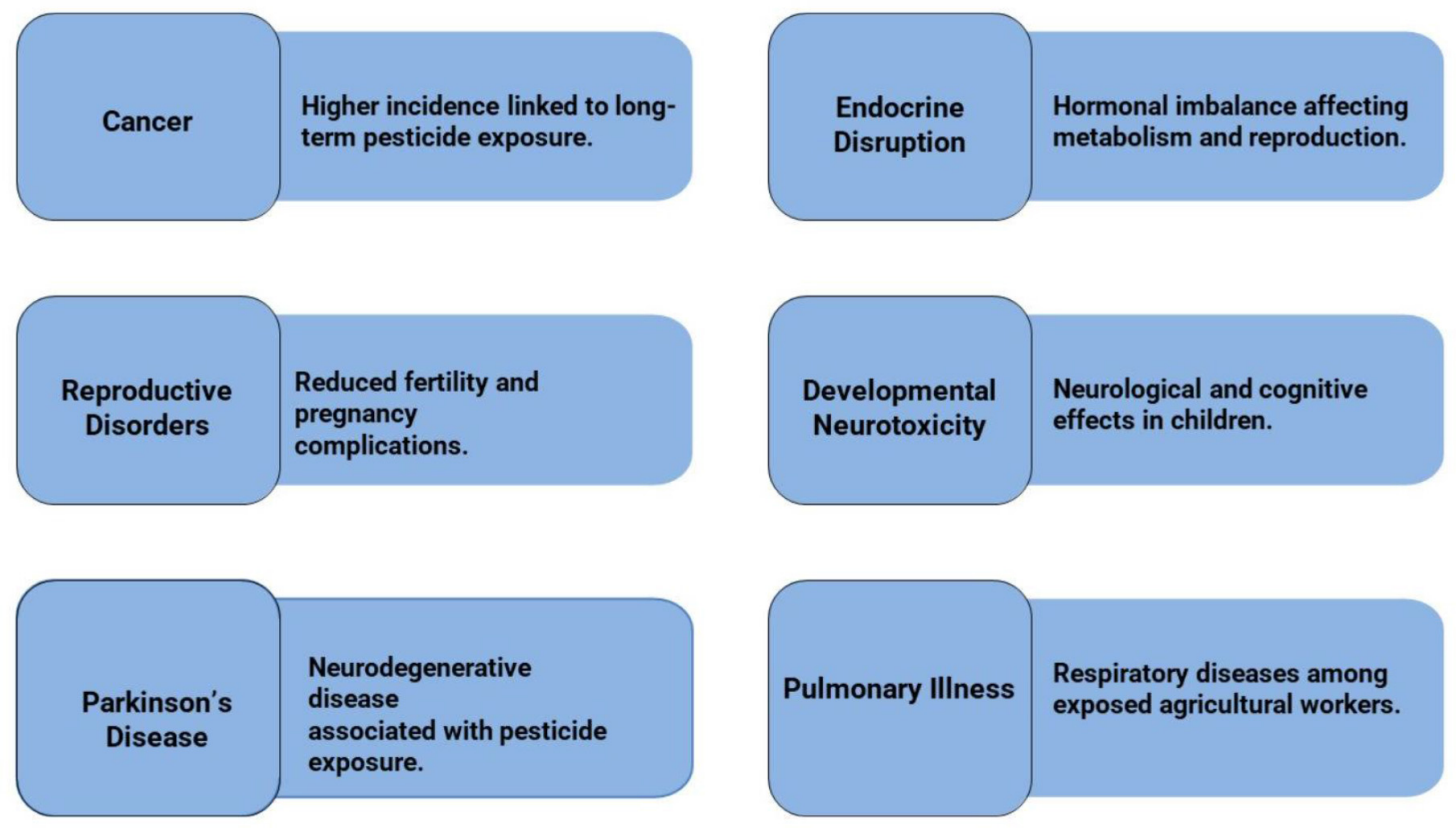
Human health outcomes associated with chronic pesticide exposure.

Several publications have proved that overuse of pesticides has led to food chains and environmental compartments contamination, resulting in biodiversity loss and chronic human exposure (14, 15). Chronic exposure has been associated with higher risks for cancer, endocrine disruption, reproductive disorders, developmental neurotoxicity in children, selenium transport deficits and Parkinson's disease, as well as pulmonary illness especially among farmers (Figure 2). These results underscore the urgent requirement for environment-friendly options that use minimal chemical pesticides yet ensure efficient crop protection (42–46).

### 3.2 Fertilizers, water contamination, and nutritional quality

Chemical fertilizers have a critical role in increasing crop production; however, their excessive use also brings great potential hazards for the environment and human health. Excess nitrate in soil and groundwater harms the safety of drinking water and can have large disparate impacts on susceptible populations, including fetuses (16). Health effects related to nitrate exposure were methemoglobinemia, thyroid dysfunctions, adverse pregnancy outcomes and raised the risk of cancer through nitrosamine formation (17). Recent studies show that nitrate exposure, mainly from contaminated drinking water, can pose serious health risks beyond the well-known—but now less common—infant methemoglobinemia. Even nitrate levels below current regulatory limits (10 mg/L nitrate–N or 50 mg/L nitrate) can be harmful because nitrate is converted to nitrite, which can then form carcinogenic N–nitroso compounds. Evidence links drinking water nitrate, particularly at levels of 9.25 mg/L or higher, to an increased risk of colorectal cancer, with some studies reporting a 1.47-fold higher risk. Elevated nitrate in groundwater has also been associated with thyroid problems, especially in young adults, because nitrate competes with iodide uptake, potentially leading to hyperthyroidism and, over time, thyroid cancer. In fact, up to 97% of human exposure to N–nitrosamines (N–NAs) comes from this internal conversion of nitrate to nitrite to N–NAs, a process that is worsened by low intake of antioxidants, like vitamins C and E, and high consumption of red meat (16, 18, 19).

Nutrient pollution leads to eutrophication, reduced oxygen levels in water bodies, and loss of biodiversity, thereby compromising the ecosystem services essential for food security (47). Based on this, organic fertilizers and biofertilizers are sound options due to the agro-ecological alternatives, which promote deleterious soil effects with respect to land (3).

### 3.3 Antibiotic use and antimicrobial resistance

Zoonotic pathogens account for 75% of emerging human infections and pose a major global threat to animal health, human health, and food security (20). The extensive use of antibiotics in agriculture, particularly in intensive livestock and aquaculture systems, represents a critical threat to global public health. Antibiotics have historically been used not only therapeutically but also for growth promotion and disease prevention, practices that have accelerated the emergence and dissemination of AMR across food chains, environmental reservoirs, and human populations (21, 22). AMR is now recognized as one of the most pressing global health challenges, responsible for over one million direct deaths annually, with projections indicating up to 10 million deaths per year by 2050 if current trends persist (23, 24).

In response, international organizations have strengthened regulatory frameworks to restrict routine antibiotic use in agriculture. The World Health Organization and the European Union have implemented measures to limit prophylactic use and protect critically important antimicrobials, while promoting biosecurity, vaccination, improved husbandry, and surveillance systems as sustainable alternatives (13, 25). It becomes increasingly crucial to involve multiple sectors in order to detect and respond to zoonotic dangers, both present and emerging. As a result, several nations now concentrate on enhancing intersectoral cooperation and disease preparedness within the context of the “One Health” strategy (26).

As aquaculture continues to grow as the fastest-growing sector of food production, antimicrobial resistance (AMR) is becoming a greater global problem. Antimicrobials are crucial for preserving fish health and productivity, but when they are misused or handled improperly, they can pose major health concerns to people all around the world. Aquaculture's ongoing misuse of antibiotics has increased microbial community selection pressure, which has led to the establishment and spread of resistant strains that endanger public health, ecological integrity, and food safety. Due to this, AMR in aquaculture is receiving international attention and calls for science-based management techniques, sustainable innovation, improved cooperation, and responsible antibiotic usage (27, 28).

## 4 Integrative frameworks: One Health and planetary health

One Health is an interdisciplinary approach that aims to address health issues at the interface of humans, animals, plants, and ecosystems. It calls for a holistic and systems-based approach that recognizes the interconnection between the health of humans, animals, plants and the environment. Recognition of the interconnected nature of these challenges has driven the adoption of the One Health framework, which acknowledges the fundamental interdependence of human, animal, plant, and environmental health. Defined as an integrated, unifying approach to optimizing health across these domains, One Health promotes multisectoral collaboration to address shared threats such as zoonotic diseases, AMR, and ecosystem degradation (29, 30). Approximately 75% of emerging infectious diseases are zoonotic in origin, driven largely by anthropogenic pressures including habitat destruction, intensive agriculture, wildlife trade, and climate change (31).

Operationalization of One Health principles is reflected in global initiatives such as the Quadripartite One Health Joint Plan of Action (2022–2026), which emphasizes integrated surveillance, early warning systems, and coordinated responses at the human–animal–environment interface (31). Collaborative research under this framework enhances outbreak detection, source attribution, and risk assessment along the farm-to-fork continuum, thereby reducing foodborne disease incidence, economic losses, and AMR emergence (32).

Complementing One Health, the planetary health framework broadens the perspective by explicitly linking human health to the stability of Earth's life-support systems. By focusing on planetary boundaries, climate change, biodiversity loss, and pollution, planetary health addresses the systemic drivers of health inequities and environmental degradation (33, 34). Increasing convergence between One Health and planetary health is advocated to address the “triple planetary crisis” through transdisciplinary research, policy coherence, and transformative societal change (35).

## 5 Regulatory challenges within the One Health framework

The unregulated and reckless use of synthetic fertilizers, pesticides, and other dangerous chemicals is a major but mostly avoidable hazard to human wellbeing, animal health, and environmental sustainability within the One Health paradigm. Therefore, there is an immediate need for policy action to curb the indiscriminate use of these inputs by adopting regulatory frameworks that restrict highly hazardous chemicals and require thorough risk assessments through all pathways of environmental and human exposure as well as improved monitoring systems and enforcement mechanisms (36, 37). Bringing agricultural policies into line with environmental and public health policies under a One Health approach through stricter residue monitoring, transparent labeling, protective exposure limits, and robust surveillance of pesticide-related illnesses will be key to reducing chemical burdens and environmental contamination (38, 39). At the same time safe sustainable alternatives should be promoted such as integrated pest management organic farming agroecological practices that can help in slowly moving away from over-reliance on petrochemical-based agricultural inputs but keeping ecosystem resilience intact for public health today as well as tomorrow (40, 41).

## 6 Discussion: future directions and policy implications

Policy coherence across health, agriculture, environment, trade, and labor sectors is critical in order not to have contradictory incentives and regulatory gaps that sustain injudicious chemical use as well as AMR. Fragmented governance can lead, for instance, to pesticide registration policies and subsidy policies that are against public health goals or agricultural productivity targets that do not take into account environmental limits. Aligning regulations standards and fiscal policies with One Health goals such as restricting highly hazardous pesticides phasing out non-therapeutic antimicrobial use in livestock plus rewarding low input biodiversity friendly farming creates a more enabling environment for sustainable practices. Coherent policies also facilitate cross border coordination in regions where trade plus transboundary ecosystems link food systems plus health risks.

Frameworks like the Quadripartite collaboration (FAO, WHO, WOAH, and UNEP), create formal pathways for knowledge sharing, joint training, aligned guidance, and coordinated governance that can help support a One Health approach. Integration of environmental and agricultural aspects into health-dominated agendas—and the reverse—is facilitated by such collaborations through shared technical documents, joint situation assessments, and country support. They also have room to support investment frameworks that would allow the flow of funds toward interventions with clear co-benefits for health, climate, biodiversity and livelihoods. For example: safer pest control strategies; better veterinary and plant health services; infrastructure in food systems that are resilient.

In the end, food systems need to be seen as “chains of life” situated within planetary boundaries instead of just economic supply chains that are made better by increasing volume and profit in the short term. This new view puts more importance on keeping ecological functions working well, soil staying healthy, water being clean, and genetic diversity existing as very important conditions for productivity and health over the long term. It brings out very clearly that real food costs include things like pollution, disease burdens, and loss of biodiversity which are usually paid by marginalized rural communities and future generations.

This kind of change puts regenerative agriculture, equity, and health-promoting diets at the heart of policy and investment choices. Regenerative practices that heal soils, store carbon, and increase landscape diversity can cut back on the need for synthetic inputs while improving resilience to pests, diseases, and climate shocks. Encouraging diverse, minimally processed, plant-rich diets can lower the demand for high-input monocultures and intensive livestock systems linked to deforestation, pollution, zoonotic risks, and AMR. Putting equity considerations—like fair access to land resources and decision-making power for smallholders, women and Indigenous peoples—into practice ensures that the advantages as well as the disadvantages of transition are more justly shared hence increasing social legitimacy and political durability of reforms.

All these things add up to what is an imperative paradigm shift from chemically intensive narrowly productivity focused agriculture toward integrated health-oriented food systems that operate within ecological limits. This shift is not just a technical option but a societal and political one; it requires long-term commitment cross-sectoral governance as well as meaningful participation from those most affected by current and future health risks.
